# Effects of a Physical Exercise Program (PEP-Aut) on Autistic Children’s Stereotyped Behavior, Metabolic and Physical Activity Profiles, Physical Fitness, and Health-Related Quality of Life: A Study Protocol

**DOI:** 10.3389/fpubh.2018.00047

**Published:** 2018-03-02

**Authors:** José Pedro Ferreira, Chrystiane Vasconcelos Andrade Toscano, Aristides Machado Rodrigues, Guilherme Eustaquio Furtado, Mauro Gomes Barros, Rildo Souza Wanderley, Humberto Moreira Carvalho

**Affiliations:** ^1^Sport and Physical Activity Research Center, University of Coimbra, Coimbra, Portugal; ^2^Federal University of Alagoas, Maceió, Brazil; ^3^Polythecnic Institute of Viseu, Viseu, Portugal; ^4^State University of Pernambuco, Recife, Brazil; ^5^Federal University of Santa Catarina, Florianopolis, Brazil

**Keywords:** exercise, intervention studies, autism spectrum disorders, stereotypes, quality of life, biomarkers

## Abstract

Physical exercise has shown positive effects on symptomatology and on the reduction of comorbidities in population with autism spectrum disorder (ASD). However, there is still no consensus about the most appropriate exercise intervention model for children with ASD. The physical exercise program for children with autism (PEP-Aut) protocol designed allow us to (i) examine the multivariate associations between ASD symptoms, metabolic profile, physical activity level, physical fitness, and health-related quality of life of children with ASD; (ii) assess the effects of a 40-week exercise program on all these aspects of children with ASD. The impact of the exercise program will be assessed based on the sequence of the two phases. Phase 1 is a 12-week cross-sectional study assessing the symptomatology, metabolic profile, physical fitness and physical activity levels, socioeconomic status profile, and health-related quality of life of participants. This phase is the baseline of the following phase. Phase 2 is a 48-week intervention study with a 40-week intervention with exercise that will take place in a specialized center for children with ASD in the city of Maceió-Alagoas, Brazil. The primary outcomes will be change in the symptomatic profile and the level of physical activity of children. Secondary outcomes will be anthropometric and metabolic profiles, aerobic function, grip strength, socioeconomic status, and health-related quality of life. The study will provide critical information on the efficacy of exercise for children with ASD and help guide design and delivery of future programs.

## Introduction

Autism spectrum disorder (ASD) refers to a complex category of the neurobiological development disorders, which is typically diagnosed during childhood (1). The main symptomatological characteristics are (a) persistent deficits in social communication and social interaction and (b) restricted, repetitive patterns of behavior, interests, or activities. Symptoms may present in three levels of intensity that is directly related to the necessary support: level 3 requiring very substantial support, level 2 requiring substantial support, and level 1 requiring support in the main areas of symptomatic ASD (2).

Estimates of the prevalence of ASD are scarce (3, 4) possibly due to the complexity of the symptoms and comorbidities associated with the disorder (5). To the best of our knowledge, the only study carried out in Brazil on the prevalence of ASD (6) showed that prevalence was almost 0.3% in school age children, much lower than figures reported in most other surveys (7). The majority were males (80%) confirming the 4:1 male/female ratio reported (8, 9) and born from older mothers (50%), consistent with recent studies documenting parental age as a risk factor for autism (10).

In addition to studies aiming to reduce the symptomatic effects (11), attention has been paid to the health of the child with ASD (5, 12, 13). Several studies have suggested that children with ASD present greater health problems when compared to children with normative development (14, 15). There is evidence that children with ASD have higher risk of comorbidities (15, 16), sleep disturbance (17, 18), metabolic disorders (19), hyperactivity (20, 21), motor activity disorders (22), obesity (23–26), and lower health-related quality of life for children and their families (5, 12).

Autism spectrum disorder symptomatological characteristics cause significant interference in physical activity (PA) patterns (27, 28). Deficits in social communication are barriers that limit access to sports in school and to free time playing experiences after school (29–31). Also restricted and repetitive patterns of behavior limit an individual’s interaction with the environmental context because this pattern is involuntary, with an exclusive function of producing physical and sensorial self-regulation (32).

It is more likely that children with ASD show higher deficits in motor abilities when compared to children without ASD. There is evidence showing that motor deficits in children with ASD are characterized by balance, postural stability, coordination deficits, and presence of motor dyspraxia (22). Children with ASD are less active than children without ASD, observed when comparing time of moderate and vigorous physical activities (33–35). Thus, children with ASD are more vulnerable to overweight and obesity (33, 36–38).

Interventions have been conducted to reduce ASD symptoms (11) and comorbidities (12, 13). Physical exercise (PE) as a treatment, for example, started in the early 1970s and showed positive effects on aquatic skills and social interaction learning (39). Currently, systematic reviews (40, 41) and meta-analyses (42, 43) have shown positive effects for PE on both symptoms and comorbidities related to the disorder with the strongest evidence for symptom improvement when compared to improvements in the individual’s overall health (44).

Among the positive effects reported as a result of PE are a reduction in stereotyped (45, 46) and aggressive behavior (45) and a decrease in body mass index (BMI) in children with ASD (47). In addition, improvements have been seen in motor coordination, dynamic equilibrium (22, 48), manual muscle strength (49), academic performance (45, 47), and different psychosocial domains (50). Detailed information provided in these papers on the specifics of exercise interventions for children with ASD, such as exercise intensity, volume, and frequency, has largely been absent (41). Unfortunately, this has made replication difficult, and the identification of effective exercise components has not been possible (42). Walking and running programs are the most common modes of delivery (47, 51–53), followed by water-based activities (39, 49, 54–56). Few studies have reported the intensity of PE (44). Two studies reported attempts to control exercise intensity to a range of 50–60% of the predicted maximum heart rate (PMHR) with a progressive increase to 70–80% of PMHR (54, 57). The length of the PE interventions has ranged from 8 to 36 weeks, with a session frequency of two to three times per week and a session duration of 20–40 min (44).

There is also limited description of any special adaptations used in the program for individuals with ASD (41, 42, 46) since it is possible that they do not process exercise in the same way as in individuals without ASD (44).

In the existing literature, there is a shortage of specific information such as age (58–60) and diagnostic variations of the participants (49, 54, 57, 61) making comparison between studies less meaningful. From a research perspective, insufficient details on the preintervention and postintervention evaluation procedures (41, 42, 46) or the measurement instruments used (39, 51, 52) are provided to allow replication.

Therefore, there is the need for an intervention study with a design that specifies the characteristics of the exercise program, the adaptation procedures, the different intervention phases, and the type of assessment used, so that a closer understanding of the specific effects of exercise on symptoms and comorbidities can be achieved.

## Aims of the Study

(a) To examine the multivariate associations among ASD symptoms, metabolic profile, PA level, physical fitness, and health-related quality of life of children with ASDs.
(b) To assess the effects of a 40-week exercise program on the metabolic profile, PA level, physical fitness, and health-related quality of life of children with ASD.

## Initial Procedures

### SEC-Aut Eligibility Criteria

During the last decades, the WHO has increasingly recognized the role of exercise and PA and its impact on health and well-being, including individuals with disabilities. Thus, institutions, families, and caregivers are particularly receptive to participate in exercise intervention programs, particularly in social contexts and communities where the offer for such services is scarce or inexistent. Four specialized education centers (SEC-Aut) in the city of Maceió, Alagoas-Brazil, will be formally contacted to confirm their expressed interest and availability to potentially participate in this study and create the singular conditions required for its implementation with children with ASD. From the four SEC-Aut’s assessed, only one will be selected to take part in the study to ensure that all children with ASD participating in PEP-Aut are experiencing the same attendance procedures, type of intervention, weekly training volume, and the same type of exercise setting. The three excluded SEC-Aut’s will alternatively participate in 4-month recreational and sociocultural activities program (1 day per week) offered by a member of the research team at the Department of Physical Education of the Federal University of Alagoas, Brazil. The SEC-Aut will be selected using the following eligibility criteria: (i) availability to participate in all phases of the study during a total period of 22 months; (ii) potential for signing up the largest number of participants eligible for the study; (iii) existence of an indoor and outdoor area with appropriate conditions to implement PEP-Aut sessions; (iv) adequate professional and family support to implement and monitor the PEP-Aut sessions in children with ASD; and (v) willingness to work with the research team on adjustments and improvements in the intervention protocol.

An information session will take place in the selected SEC-Aut, where participants and their parents and legal representatives will be informed about the study’s specific objectives, data collection procedures, and characteristics of the intervention program. This study will follow the recommendations of ethical guidelines for research with human beings (62) and the Brazilian Health Council Resolution, number 466 of December 12, 2012, with written informed consent from all subjects (63). Written consent was obtained from the participants and their parents in accordance with the Declaration of Helsinki (64). The protocol was approved by the Federal University of Alagoas Ethical Committee (Reference number: 1.091.864).

### Participants with ASD Eligibility Criteria

The eligibility criteria for ASD participants include the following: (i) to have a certified medical diagnosis of Asperger’s syndrome, autism, or developmental disorder without specification, in accordance with the standards established in the Diagnostic and Statistical Manual of Mental Disorders, fifth edition (DSM-5) (1); (ii) to present a recent history of non-participation in PE programs or similar physical motor activities; (iii) initial medical checkup to determine fitness to participate in PE program; and (iv) the absence of other syndromes and motor impairment conditions associated with ASD.

## Methods/Design

**Figure 1:**
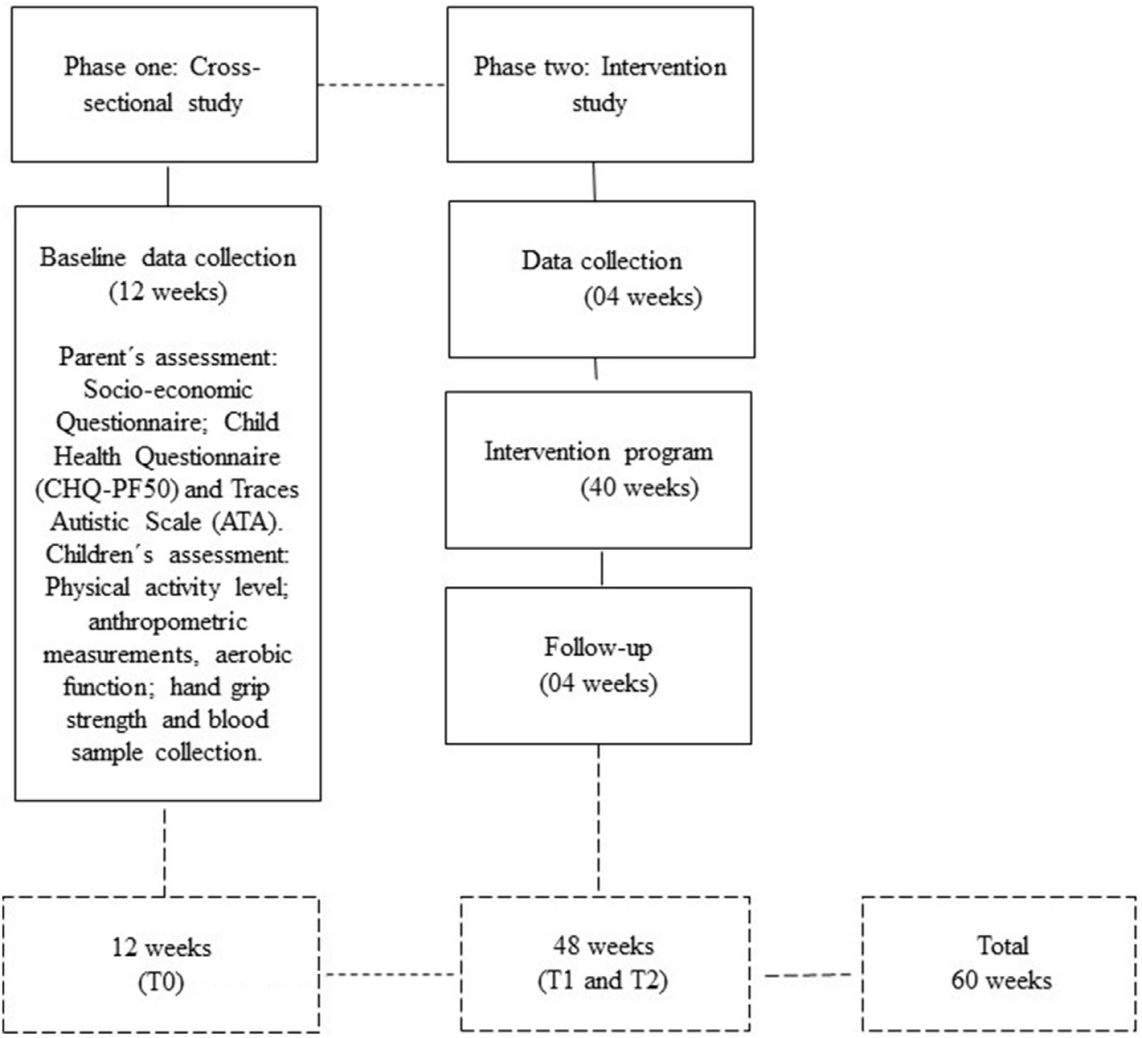
Timeline of the PEP-Aut’s phases (cross-sectional and intervention).

This research will take approximately 60 weeks and will run over two different phases (see Figure 1):

(i) Phase 1: a cross-sectional study, consisting of 12 weeks, with the assessment of a large number of children with ASD (*N* = 145) from different SEC-Auts, in the city of Maceió on anthropometric (body mass, height, BMI, waist circumference and triceps, biceps, gemstone, subscapular, suprailiac, and abdominal skinfolds), aerobic function (1 mile run/walk test), hand-grip strength, and blood sample variables (glucose, total cholesterol, triglycerides, HDL, and LDL). Different sociometric parameters such as the child’s state of health and the profile of autistic traits will be assessed using questionnaires administered to parents or legal representatives.
(ii) Phase 2: an intervention with exercise that take place over 48 weeks, including 40 weeks of PE (PEP-Aut) and 8 weeks of assessment (4 weeks before and 4 weeks after the intervention). We will simply randomize children with ASD into two groups, one exercise group and one control group. The control group will maintain daily regular activities, but will not be participating in any type of PE.

## Estimated Sample Size

The variable “occurrence of stereotyped behaviors” will be the primary outcome of this study. We will calculate the total sample from the power associated with the effect size and the difference of the means from the overall effect size of the results from similar studies that test the effectiveness of the PE programs in children’s stereotyped behaviors, clinically diagnosed with ASD (Cohen’s d effect size = 1.41) (45, 52, 53, 60).

To detect possible differences between the means of the primary measure with a power of 80% (*n* = 0.05), 39 children would be necessary to verify a positive effect of the PE program on the stereotyped behaviors of this population. In addition to these children, and aiming to prevent possible losses estimated as 30% characteristic from longitudinal PE programs, 12 participants will be added to the initial sample, and totally 51 individuals will be selected for the study (65).

For the Phase 1 cross-sectional study, it is possible to assess at least 145 participants from both sexes, with age ranging from 4 to 12 years, following a procedure reported in recent studies (40, 43).

## Program Attendance/Adherence

The participants will be asked to attend the PEP-Aut sessions twice a week, during the regular attending hours at the SEC-Aut, for a period of 48 weeks. The intervention with PE will last for a total of 40 weeks, including a total frequency of 80 exercise sessions and 4 weeks of preevaluation and 4 weeks of postevaluation. Attendance will be recorded, and in the case of two consecutive absences, we will use the following procedures: (a) telephone contact, made by institutional social services, to parents or legal guardian to identify reasons related with the absences; and (b) home visit to identify reasons related with the absences. There was no guidance from previous studies on the expected percentage of adherence to PE intervention programs in children, adolescents, and adults with ASD (44), so a cutoff value for inclusion of participants in final statistics will be determined following the attendance rates seen in the pilot study.

## Masking

Blood samples collection and global health assessment will be performed by a registered nurse and a certified medical doctor. The anthropometric and physical fitness assessment will be performed by a certified member of the research team with expertise in both exercise and services provided to children and youngsters with ASD. The administration of the questionnaires to parents and legal representatives, assessing the socioeconomic status, the health-related quality of life, and the administration of the Scale of Autistic Traits (SAT) and the Childhood Autism Rating Scale (CARS) will be organized by the main investigator and will be performed together with a certified psychologist working with the research team. To minimize differences in the procedures, the same evaluators will perform data collection in the different assessment moments over the study. Each member of the assessment team will collect the same specific data, will not make any reference to the exercise program, and will not have any access to the remaining data.

## Outcome Measures

All outcome measures will be collected two times: (a) in Phase 1 (cross-sectional study, weeks 1–12) and (b) in Phase 2 (intervention study, between weeks 13–16 and 57–60). Primary outcomes are related to the symptoms profile and the level of PA. Secondary outcomes will be the metabolic profile, the anthropometric measurements, the functional level, the handgrip strength, the socioeconomic status, and the health-related quality of life. A characteristic of this study is that some of the measurements are applied to participants families or legal representatives (Socio-economic Questionnaire, Child Health Questionnaire, SAT, and CATS), while others are applied to the participants with ASD themselves (PA level, anthropometric measures, aerobic function, hand-grip strength, and blood collection).

## Assessment of Parents and Legal Representatives

### Socioeconomic Status

A questionnaire developed by the Brazilian Association of Market Research Institutes, version 2012, which is based on the family budget survey of the Brazilian Institute of Geography and Statistics (IBGE), will be used to estimate the purchasing power of individuals and families and to define economic classes. The questionnaire considers consumption patterns or potentials (television, radio, bathroom, automobile, house cleaner, washing machine, video cassette and or DVD, refrigerator, and freezer), and level of schooling of the head of the family (from illiteracy to higher education level complete). Each item is classified using a Likert-type scale (0–4) with specific punctuation for type and number of items. The sum of the items (0–46 points) allows the definition of the economic class (A1, A2, B1, B2, C1, C2, D, and E), where A1 represents the highest economic status, monthly income of R$12,926, and E represents the lowest economic status attributing monthly income of R$477, lower than the Brazilian minimum wage (66).

### Child Health Status

We will use the Child Health Questionnaire (CHQ-PF50), which is a generic instrument to assess the quality of life related to physical and psychosocial health in childhood from the parents’ perception (67). The instrument has 50 items organized in 15 domains: physical capacity, social role of limiting daily activities due to emotional and behavioral aspects, social role of limiting daily activities due to physical capacity, body pain or discomfort, behavior, mental health, self-esteem, health status, family impact, and family cohesion (68). The score of each domain is presented on a scale of 0–100, with the highest score representing the best state of health, well-being, and satisfaction. Additional information on this instrument is available at https://www.healthactchq.com/.

We will use the Brazilian Portuguese version of the CHQ-PF50 (68). The questionnaire was translated and validated in the context of the Brazilian population with chronic disease. The validation study reported Cronbach’s alpha values above 0.70 in 10 (91%) of the 11 measurable health concepts (i.e., health concepts with more than one item) of CHQ (total 0.94, range, 0.40–0.94), except for the perception of health status (0.40). Reliability based on the repeated measurements within an average of 8.5 days showed intraclass correlation coefficients with an overall mean of 0.60 (0.20–0.90) for the 15 health concepts evaluated by the CHQ, thus acceptable reproducibility. The concepts with the lowest reproducibility were those related to the social role of limiting daily activities due to physical capacity, perception of health status, change in health status, and emotional impact in the family.

### Childhood Autism Rating Scale

Childhood Autism Rating Scale is a standardized instrument to identify levels of intensity of ASDs (mild, moderate, and severe), as well as the sharp distinction between autism and intellectual disability (69). The instrument is based on the diagnostic criteria of Kanner (70), Creak (71), Rutter (72), Ritvo and Freeman (73), and DSM, third edition (DSM-III). The Brazilian Portuguese version of this instrument (CARS-BR) (69) will be used to evaluate the child’s behavior from 14 domains that are usually affected by serious problems in autism, plus a general category of autism impressions. The aim of the instrument is to identify children with ASD and differentiate them from children with other developmental disorders. The 15 items of the scale are relative to people, imitative behavior, emotional response, body use, use of object, adaptation to change, visual response, auditory response, perceptual response, fear or anxiety, verbal communication, non-verbal communication, activity level, level and consistency of intellectual relations, and general impressions. The score ranges from 1 to 4 for each item: 1 indicates adequate behavior for the age level, while 4 indicates severe deviation from normal behavior for the age level. The scores of the individual items are summed in a total score, which rates the child as non-autistic (below 30), mildly or moderately autistic (30–36.5), or severely autistic (above 36.5). It is also necessary to count the number of items in which the child scored at or above 3. A diagnosis of severe autism is appropriate if these items are at least 5. A score of 30 or more is used as the threshold value for diagnosis of autism.

The validation process was attended by 60 patients and, in terms of validity, demonstrated a very good internal consistency, with mean values of Cronbach’s alpha coefficient of 0.82 (95% CI, 0.71–0.88), indicating a high degree of internal consistency (69, 74). Data on the convergent validity, compared to the Autistic Traits Assessment Scale, showed Pearson’s correlation coefficient values with *r* = 0.89. The test–retest reliability had a kappa coefficient value of 0.90.

### Scale of Autistic Traits

The SAT is an instrument that allows a first identification of the symptoms profile of ASDs (75). In its original construction, the diagnostic criteria of DSM-III, DSM-III-R, and ICD-10 were considered to develop the instrument. The ATA scale is an easy-to-apply instrument, accessible to professionals who have direct contact with the autistic population (e.g., teachers or even parents) informing the current state of the patient. The instrument was developed to be used by professionals, not necessarily medical doctors, responsible for the evaluation of the answers according to each item. It is not, therefore, a diagnostic interview, but a standardized test that gives the cognitive behavioral profile of the child with ASD. The scale score is based on the following criteria: (i) each subscale of the test has a value from 0 to 2; (ii) the positive scale is scored when one of the items is positive; (iii) the overall scale score is based on the arithmetic sum of all the positive values of the subscale, allowing a very precise characterization of autism.

The Brazilian Portuguese translation and validation considered corrections of the diagnostic criteria, resulting from the publication of the DSM-IV (76). The cutoff point was 15 (*p* ≤ 0.05); the coefficient of variation (reliability) was 0.27, and the external validity showed low agreement (kappa = 0.04). Finally, the internal validity was 100%, showing that the clinical diagnoses agreed with the results obtained by applying the scale. The correlation value obtained was 0.42, being specific for the autistic conditions. It also presented good internal consistency, with Cronbach’s alpha 0.71. Thus, the scale is considered to be adequate and reliable for us to use in our applied educational context, with children and young people involved in PE.

### Assessment of Children’s PA

ActiGraph tri-axial accelerometers will be used to assess the children’s PA level. The accelerometer allows the recording of body acceleration in the lateral, vertical, and anteroposterior axes, and their respective magnitude vectors. We will program the accelerometers to record epochs at each second, and subsequently, in the data reduction phase, the epochs will be grouped into blocks of 15 s. A consecutive period of 90 min without any record of body acceleration is considered as non-use time of the apparatus and will be excluded in the data reduction phase (77). The cutoff points adopted for the determination of PA intensities are sedentary (equal to or less than 1,099 cpm); light (1,100–3,199 cpm); moderate (3,200–8,199 cpm); and vigorous (equal to or greater than 8,200 cpm) (78, 79).

### Anthropometric Measures

Anthropometric measures will take place separately, in a private room, following standardized procedures (80). Data collection will include a set of simple anthropometric variables such as total body mass (kilograms); stature (centimeters); BMI (kilogram per square meter); waist circumference (centimeters); and triceps, biceps, calf, subscapular, supra iliac, and abdominal skinfolds (millimeter). Body mass (kilograms) will be determined with a portable digital scale (Seca^®^, Model 770, Birmingham, UK). Stature (centimeters) will be determined with a portable stadiometer (Seca^®^, Model 206, Birmingham, UK) with a precision of 0.1 cm, waist circumference (centimeters) will be measured with an anthropometric tape (Seca^®^, Model 201, 205 cm) with a precision of 0.1 cm, and skinfolds will be measured with an adipometer (millimeter) (Model OPUS^®^ Max 30).

### Aerobic Function Measures

The assessment of aerobic function will be achieved through the 1 mile run/walk test protocol from the Fitnessgram Test Manual (81). The purpose of this test is to complete one mile in the fastest possible time. If the participant desires, walking may be interspersed with running; however, he/she should be encouraged to cover the distance in as short a time as possible. For the particular case of younger children, modifications should be introduced and a shorter test will be performed; 1/4 mile for 6–7 years old and 1/2 mile for 8–9 years old, using similar procedures to the original test protocol.

### Hand-Grip Strength Measures

Manual gripping force (kgf) will be assessed using a SAEHAN^®^ Hydraulic Hand Model SH5001 dynamometer. The position used for the data collection is the one recommended by the Brockport Fitness Test Manual (82).

### Blood Sample and Analysis

Blood will be collected by venepuncture, in a fasting state, by an independent registered nurse and will be analyzed using a standard protocol (83) by a previously defined certified laboratory. Individual results are sent to the research leader in a printed format and without any storage of the biological material collected. Children’s parents or legal representatives will receive a copy of the individual lab test results containing individual information about the child’s metabolic profile (glucose, total cholesterol, triglycerides, HDL, and LDL).

## Characterization of the Physical Exercise Program (PEP-Aut)

Based on a preliminary pilot study (see Supplementary Material), the design of PEP-Aut was build and tested following recommendations from summaries of the existing literature where exercise has been used with children with ASD (22, 48, 84). In addition, we used other relevant guidelines for exercise intervention programs in the general pediatric population (85).

### Adaptation (4 Weeks)

The purpose of this phase is to enable children with ASD to adjust their behavior to the characteristics of the different exercises and tasks included in PEP-Aut (see Table 1). Adaptive sessions will be mediated by parents or legal representatives and supervised by the researcher.

**Table 1:** Overview of the PEP-Aut specific physical exercises used for intervention.

Exercise	Physical ability	Description	Resource
Climbing and support in the bar	Upper limb strength	The child should climb a vertical backrest, reach the last bar, and hold the body suspended for 5.0 s	One standard vertical backrest with 1.5 m height, fixed at 0.5 m of the ground
Release to the basketball	Upper limb strength	Starting from an initial position with a mini medicinal ball close to the chest, the child should perform a shoulder lift (180°) followed by an elbow flexion, positioning the mini ball over the head. From this position, the child should then do a full extension of the upper limbs (elbow and forearm) followed by a slight flexion of the wrist, performing the ball throwing movement	One basketball table (fixed at 1.75 m from the floor), three benches to support the throwing with different dimensions (base 0.5 cm × height 0.5, 1.0, and 1.5 m); Mini medicinal balls with different weights (0.5, 1.0, and 2.0 kg)
Elastic bands workout	Upper and lower limbs strength	The child in an upright position, with the arms suspended along the body, picks up the elastic bands, which should be fixed to the floor by a safety beam, by the fists. The child should perform a simultaneous flexion of the forearms, bringing the hands closer to the shoulders for each repetition	Elastic bands
Climbing the steps and walking on the inclined plane	Lower limbs strength and coordination	The child should climb the three steps and walk on the inclined plane (hip and knee flexion movement)	l-shaped wooden staircase with three steps (0.12 m × 0.15 m) and inclined plane of 0.78 m length and 0.30 m height with handrail throughout the entire length
Step box with target	Lower limbs strength and coordination	The child should climb three sets of sequenced steps. When reaching the last step, he/she should perform a plantar flexion of the ankle and try to reach the target fixed to the wall above his/her head and score points	Six steps with the dimension of 0.60 m × 0.28 m × 0.14 m overlapped and placed in ladder. The first step consists of a unique step, the second step consists of two sets of overlapping steps, and the third step consists of a set of three overlapping steps, respectively
Sequenced march	Coordination	The child should perform front running on a sequence of five arcs arranged sequentially in the ground	Five plastic bows with 0.50 m in diameter

### Intervention (40 Weeks)

After the adaptation to the exercises, the PEP-Aut sessions will have the following structure:

(a) Preparatory phase (5 min)—Time period where ASD children are prepared for the exercise session, including active search, displacement of the children and their parents/legal guardians to the transition area, placement of the heart rate monitor and of the leg and ankle weights, and transfer of the child to the PEP-Aut working area (indoor or outdoor).
(b) Development phase (30 min)—Time period where children perform a brief warm up and perform strength, balance, and coordination exercises in a training environment. The sequence of the exercises in the program is not rigid to maintain high levels of interest and fun. Different motivational strategies were used to maintain motivation and adherence to the PEP-Aut including using different materials, different colors, music in some sessions, and small rewards. Such strategies may contribute to reduce or eliminate barriers during the sessions influencing the participation and permanence of the child in the sessions.

The equipment needed to implement the PEP-Aut sessions will be presented simultaneously in the working space using a circular organization. The type of communication employed with each child will be determined in consultation with parents/legal representatives. We will adopt three levels of mediation: (a) oral explanation of the exercise facing the child, (b) oral explanation with subsequent modeling, and (c) oral explanation, modeling, and child orientation throughout the intervention process (skills development and acquisition).

(c) Return to calm phase (5 min)—After the development phase, the child should move to the transition area, remove the leg and ankle weights, and start immediately the relaxation activities. Parents and legal representatives will perform relaxation exercises using tactile slip skills on the child’s back and belly aiming to return the child to calm. These procedures will respect the child’s choice about his/her favorite position for the relaxation massage: (a) laying on the mat, (b) in a sitting position on the mat, or (c) standing still on the mat.

## Description of PEP-Aut Exercises

According to the review of literature, manipulative strength, balance, and coordination are physical qualities that are often impaired in children with ASD (22, 48). Such interference is due to its relation with stereotyped movements that may appear in different ways.

The most frequently mentioned in the literature are the movement of the hands, the nods or shaking of the arms, sudden runs, body swing backward and forward, repeated manipulation of objects and fingers movements (32). These type of movements present a direct relationship with the type of exercises selected for the PEP-Aut (see Table 1).

## Adaptations of the PEP-Aut Setting

Selecting the most appropriate environment to develop an intervention project with PE requires a previous diagnosis to identify which open and closed physical space are more likely to reduce the ASD children’s level of stress. Children with ASD are less tolerant to routine changes and are resistant to new activities. For the protocol of this study, we decided to describe not only the adaptation procedures related to the physical space used during the adaptation phase of the pilot study but also those used to form the groups and to determine the type of mediation to be used.

### Physical Space

First and second sessions will be held indoor, in a global area of 40 m^2^ divided into three fixed areas, one with 2 m^2^ as a transition area, another with 36 m^2^ as a working area, and a third of 2 m^2^ as a relaxation area. The following sessions will be held in open space in an area with similar dimensions. The results of the adaptive phase of the pilot study will allow the identification of the most adequate physical space to implement PEP-Aut, for each child, and assess the possibility to use one or two types of physical space to implement the program.

### Group Environment

PEP-Aut sessions should probably function with ASD children in pairs or trios, escorted by the parents/legal representatives with the aim of building a positive motivational and socialization environment and reducing the symptoms of the disorder. Thus, the identification of each pair or trio will depend on (a) individual analysis of the child’s symptom profile based on “social interaction difficulty,” “hyperactivity/hypoactivity,” and “inappropriate reactions to frustration” variables so that pairs and trios with compatible profiles can be identified; (b) assessment of partnerships between children in both indoor and outdoor environments, with the help of parents/legal representatives; (c) not using rigid group composition, safeguarding the identification of future unproductive partnerships; and (d) children exhibiting tantrum, self- and hetero-aggression behavior, and shouting and rejection during adaptive sessions will start their PEP-Aut sessions individually and be assessed regularly about the possibility of inclusion in pairs or trios.

### Type of Mediation

During the adaptive phase, children will have a diagnosis to assess their optimal level of mediation. The major role of the mediator is to facilitate communication and interaction between the child and the PEP-Aut instructor aiming to help them to reach a voluntary relationship during the exercise program sessions and to facilitate and stimulate exploration and promote interaction in all children, particularly in those showing difficulties or refusal to participate in the exercise sessions. The literature has argued that parents, when integrated into the role of mediators in physical exercise programs, may interfere (86, 87). Thus, we will use “lack of eye contact,” “lack of attention,” and “lack of interest for learning” variables from the SAT as indicators of a better profile for the information reception offered by the mediator. In addition, we will adopt three levels of assistance that the mediator should give: (i) an oral explanation of the exercise facing the child, (ii) an oral explanation followed by a performance model, and (iii) an oral explanation, a performance model, and the child’s assistance over the different step of the exercise execution.

## Implementation of the PEP-Aut Session’s Duration

All sessions will run from Monday to Friday, in the morning (7:00–11:30 h) and in the afternoon (14:00–17:30 h), lasting for 40 min and with a maximum of three children and their parents/legal representatives. Each child will participate in two weekly sessions, on the same weekdays and schedule offered by the SCE-Aut. As an adaptive strategy for children with low tolerance to PEP-Aut sessions during the adaptive phase, we will have a progressive time participation procedure aiming not to exclude children with less initial tolerance to the exercise program. (a) In the first session, the child will participate for a minimum of 10 min, (b) in the second session, the child will participate for a minimum of 20 min, (c) in the third session, the child will participate for a minimum of 30 min, and (d) in the following sessions, the child will participate during the entire session.

## Materials Adaptations of PEP-Aut Equipment

### With Maximal Body Contact

This includes all resources whose use requires a direct partial or total physical contact with the child’s body during the sessions.

#### Shin Guard Weights (0.5, 1.0, and 1.5 kg)

The intensity (kilograms) and the volume of the working load (number of repetitions) per series will use the BMI as a reference and the symptom profile of the child, associated with the capacity to perform different levels of effort. We used the following equipment adaptation procedures: (a) on the first session, ask parents/legal representatives to make the child use at home a weightless shin guard for 1 h, for 5 consecutive days; (b) on the second session, the child will use a shin guard with a minimum mass (0.5 kg) for 40 min; and (c) in the third and fourth sessions, the child will use a shin guard with different types of loads according to the criteria established to determine the working load by age. For the number of repetitions, we used the following adaptation: (a) in the first session, the child will experience all exercises of the PEP-Aut without load and a predefined number of repetitions; (b) in the second session, the child will perform three repetitions of each one of the PEP-Aut exercises; (c) in the third session, the child will experience five repetitions of each one of the PEP-Aut exercises; and (d) in the fourth session, the child will experience seven repetitions of each one of the PEP-Aut exercises.

#### Polar RCX5 Heart Rate Monitor

The child’s heart rate will be monitored during the intervention using a heart rate monitor. We will use the following adaptive procedures: (a) in the first session, the child will use a rubber band with 8 cm wide and adjustable length on the chest for a minimum of 5 min; (b) in the second session, the child will use the same elastic band for a minimum of 15 min; (c) in the third session, the child will use the elastic band throughout the entire session; and (d) in the fourth session, the child will use a Polar RCX5 heart rate monitor throughout the entire session and register the number of resistance episodes to the equipment’s use.

### With Minor Body Contact

This includes all the equipment available to implement the PEP-Aut sessions. The equipment is presented to the participants by the parents/legal representatives aiming to stimulate curiosity and promote the equipment’s handling during the sessions.

We will use the following adaptive procedures: (a) in the first session, the child will be stimulated to handle the equipment freely (balls, bows, elastic) randomly displaced all over the session’s area. In the case of handling refusal, the mediator (parent or legal representative) should stimulate the interaction by facilitating the manipulation and the familiarization with the equipment; (b) in the second session, participants will be encouraged to explore the horizontal and vertical stairs, the step boxes, and the inclined plane. The mediator will stimulate the exploration and promote the interaction in all children particularly in those showing handling refusal; (c) in the third and fourth sessions, the participants will experience all the PEP-Aut equipment to identify a preferred exercise for each child. In the PEP-Aut sessions, and following the peak-end theory rule, all children will finish their sessions with his/her preferred exercise due to motivational reasons. In addition, individual information about the child’s ability to perform the exercises and about potential barriers will be registered and discussed with the parents/legal representatives during a meeting aiming to reduce or eliminate potential barriers and prevent a decrease on the participation and permanence of the children with ASD in the PEP-Aut sessions.

## Exercise Intensity Control

Heart rate monitors Polar RCX5 will be used during PEP-Aut exercise sessions to assess, monitor, and adjust the intensity of the heart rate interval used by children with ASD during the exercise sessions. Prescription of exercise intensity should be based on direct measurements of maximal heart rate (HR_max_) if possible, because an equation may not predict the true HR_max_ in some individuals, specific populations, or modes of exercise (88). However, in children with ASD, direct measurements are very difficult if not impossible to use due to the behavioral characteristics of the population. Thus, in this study, the exercise intensity is indirectly predicted using the Karvonen’s formula to predict target HR but with HR_max_ being calculated using the equation formula HR_max_ = 207 − 0.7 × age (89).

## Data Analysis

The distribution patterns for each variable will be assessed by visual inspection. For convenience, when needed, transformations on the variables will be made to approximate to Gaussian distributions. A multilevel modeling framework will be used, as it is a flexible approach to deal with hierarchical data, such as repeated measures, as well as the possibility to account for different levels of variation at higher orders. Particularly, multilevel modeling allows for improved estimates with repeated sampling, improved estimation with unbalanced designs, considering and modeling different patterns of variation, avoiding averaging, and retaining variation (90). Furthermore, the multilevel modeling framework is an alternative to the well-known limitation of traditional single-level-based approaches (90–95). Inspection of variation within and between clusters (i.e., groups) will be initially explored with unconditional means models, which includes only the random parameters, to measure the proportion of the total variance, which is between-participants, particularly grouped as intervention and control groups, i.e., variance partition coefficient (92). The variance components models allow the determination of whether baseline values were clustered by intervention or control group.

To examine the responses to the exercise-based intervention program in children with ASD (dummy valuable—control group coded 0, intervention group coded 1) on dependent variables, we will assume measures (level-1 unit) nested within participants (level-2 unit). A progression of two-level growth model will be used, initially considering a random intercepts models, followed with random intercepts and slopes models. Considering both variability patterns at intercept (i.e., baseline), and at slope (i.e., response to intervention) and its covariance patterns which will allow the identification of the most parsimonious model to describe the data. In addition, time variant covariates (e.g., body mass or hormone levels) and time invariant covariates will be considered for the exploration of mediating effects of responses to the intervention. To make inferences about the true (population) values of the effect of exercise-based intervention on dependent variables, the size of the SD for individual responses will be interpreted in relation to the baseline between-participant SD (96).

Validation of the multilevel models will be made by visual inspection of plots of residuals versus predicted values from the analyses. Considering available data, full maximum likelihood or restricted maximum likelihood estimations will be explored to obtain the unknown parameters. Likelihood ratio test and Akaike’s Information Criterion (AIC) will be considered for model comparisons. Multilevel models will be mostly derived using “nlme” package (97), within the R statistical language (98).

Qualitative data will be collected using a semistructured interview research format. A script of interview questions will be developed containing the thematic topics to be addressed throughout the interview, from a retrospective temporal analysis perspective as well as some key issues, i.e., the interview guide questions were inductively generated and reflected thinking, feeling, and knowing questions (99). After obtaining ethical approval, children with ASD parents and legal representatives will be invited to voluntarily agree to take part in the research, aiming to obtain a deeper understanding of their feelings, beliefs, ideas, and opinions about the effects of PEP-Aut participation in children behavior and particularly in the occurrence of stereotyped behaviors.

A voice recorder will be used to register participant’s opinions during the interview. For the analysis, each participant will have an individual identification code and a pseudonym to enable correct transcription of the verbatim audio for each interview, while guaranteeing anonymity. The interviews will be transcribed including the descriptions of non-verbal reactions of the interviewees and afterward analyzed and classified according to thematic topics/categories previously defined. To establish trustworthiness and validity, the researchers will use a triangulation method, as rigorous methods are imperative for the credibility of a qualitative study (100). Thus, different strategies will be used: (i) peer review/debriefing (i.e., first and second author will work independently at first and later will converge for data analysis and interpretation), (ii) member check (i.e., after the verbatim transcription, we will ask participants to confirm or correct the reconstruction of their statements), and (iii) rich and thick description through verbatim transcription of the all interviews (101) to bring plausibility to the data. In addition, content analysis will be used, and the most important instrument of content analysis is coding, the process of breaking down, and reducing text into manageable units of analysis (100). A semiotic analysis will be held to identify several recurring themes. To isolate the emerging thematic statements, a line-by-line analysis will be conducted, and phrases that are conceptually similar will be gathered together in categorization units.

## Discussion

This study will allow us to investigate, using both a quantitative and a qualitative research methodology, the effects of a combined physical exercise program on children’s with autism spectrum disorders symptomatology (incidence of stereotypies), metabolic and PA profiles, physical fitness, and health-related quality of life. The final goal is to develop an endurance and strength combined exercise protocol to be used to improve the health and well-being in children with ASD and promote a better quality of life for them and their families. This study also sustains the hypothetical premise that exercise reduces the incidence of stereotypic behavior (46) based on the assumption that the number of stereotypies will be reduced after participating in combined exercise sessions. The pilot study for this type of population is essential as it helps to adjust the adequacy, acceptability, and feasibility of the exercises included in the program; the adequacy of the adaptive protocol developed for children with ASD; and the extent of adherence of this particular population, up to our knowledge not tested before. This study shows a strong multidisciplinary approach, since it is prudent to assess the combined effects of exercise with some independent variables. In addition, we will also check the hypothetical premise that some objective measures have strong associations with subjective perception measures and find out what works and what does not work to enhance children’s with ASD adherence and what makes the intervention deliverable in other settings.

## Ethics Statement

This study was carried out in accordance with the recommendations of ethical guidelines for research with human beings (61) and the Brazilian Health Council Resolution, number 466 of December 12, 2012, with written informed consent from all subjects. All subjects gave written informed consent in accordance with the Declaration of Helsinki. The protocol was approved by the Federal University of Alagoas Ethical Committee (reference number: 1.091.864).

## Author Contributions

JF and AR gave a substantial contribution to the conception and design of the work, interpreted data, drafted the manuscript, and coordinated the research project. CT designed and implemented the work, collected all the data, and drafted the manuscript. HC analyzed and interpreted the data, revised the manuscript critically, and added important intellectual content. GF, MB, and RW assisted CT in the exercise program implementation, in all the data collection, revised the manuscript critically, and added important intellectual content. All authors have made a substantial contribution in their respective areas of expertise, approved the final version, and agreed to be accountable for all aspects of the work. JF and AR are CT’s supervisors.

## Conflict of Interest Statement

The authors declare that the research was conducted in the absence of any commercial or financial relationships that could be construed as a potential conflict of interest.

## Abbreviations

ASD, autistic spectrum disorder; PEP-Aut, physical exercise program for children with autism; DSM-5, Diagnostic and Statistical Manual of Mental Disorders, 5th edition; SEC-Aut, specialized education center for children with autism spectrum disorder; PE, physical exercise; PA, physical activity; HR_max_, maximal heart rate.
