# Supplementary material for: Effects of a Physical Exercise Program (PEP-Aut) on Autistic Children’s Stereotyped Behavior, Metabolic and Physical Activity Profiles, Physical Fitness, and Health-Related Quality of Life: A Study Protocol
Source: Front Public Health. 2018 Mar 2;6:47. doi: 10.3389/fpubh.2018.00047 (PMC5840149; doi:10.3389/fpubh.2018.00047)
Supplement: Supplementary file 1 [file data_sheet_1.PDF]

PREPARATION AND IMPLEMENTATION OF THE PILOT STUDY (PS): ADDITIONAL INFORMATION
(Two phases, 24 weeks)

Phase one: Preparation (4 weeks).

A systematic review was conducted aiming to identify and adapt the assessment tools, including specific adaptive procedures to enable physical activity monitoring using the accelerometers in children with ASD and minimize the risk of equipment disruption and loss of data. Phase one also aimed to choose the best measures, monitor and adapt them to produce the best test and intervention protocol minimizing the stress levels of the children with ASD.

Standard protocols with adaptive procedures were developed in phase one.

The pilot study included four weeks of initial assessment (pre-intervention), four weeks of adaptation for the children to PEP-Aut procedures, eight weeks of intervention with physical exercise using the PEP-Aut (16 sessions of 40 minutes each, twice a week, for 8 weeks) and 4 weeks of final assessment (post-intervention).

Phase two: Implementation (20 weeks).

The PS assessed the feasibility and acceptability of the PEP-Aut program to be implemented with regard to: (i) operational procedures for PEP-Aut delivery (types of exercises, equipment to control exercise intensity, equipment needed to carry out the activities and physical space); (ii) procedures to promote children´s program attendance (mediator profile, levels of help and identification of the need to reinforce participation and full permanence of the child in each session).

Identification of potential barriers that may obstruct participation and maintenance of the children with ASD in the PEP-Aut sessions.

Results provided important information for the validation of the adaptive procedures to be used in the anthropometric (Body mass, height, BMI, waist circumference and triceps, biceps, gemstone, subscapular, supra iliac and abdominal skinfolds), aerobic function (1 mile run/walk test) and hand-grip strength assessment, as well as procedure adaptations developed to stimulate children with ASD to participate in the PEP-Aut sessions.

Children were selected based on a convenience sampling method among those whose parents or legal representatives voluntarily signed the free will consent form. Not all the children involved in the pilot study will take part in the experimental study, however a motor activity program was developed to guarantee the continuity of the children's engagement in regular physical exercise.
